# Adipose-Derived Stem Cells in Cancer Progression: New Perspectives and Opportunities

**DOI:** 10.3390/ijms20133296

**Published:** 2019-07-04

**Authors:** Maria Giovanna Scioli, Gabriele Storti, Federico D’Amico, Pietro Gentile, Bong-Sung Kim, Valerio Cervelli, Augusto Orlandi

**Affiliations:** 1 Anatomic Pathology Institute, Department of Biomedicine and Prevention, Tor Vergata University of Rome, 00133 Rome, Italy; 2 Plastic and Reconstructive Surgery, Department of Surgical Sciences, Tor Vergata University of Rome, 00133 Rome, Italy; 3 Division of Plastic Surgery and Hand Surgery, University Hospital Zurich, 8091 Zurich, Switzerland

**Keywords:** cancer progression, metastasis, microenvironment, adipose-derived stem cells, ASC-based drug delivery, oncological safety

## Abstract

Growing importance has been attributed to interactions between tumors, the stromal microenvironment and adult mesenchymal stem cells. Adipose-derived stem cells (ASCs) are routinely employed in regenerative medicine and in autologous fat transfer procedures. To date, clinical trials have failed to demonstrate the potential pro-oncogenic role of ASC enrichment. Nevertheless, some pre-clinical studies from in vitro and in vivo models have suggested that ASCs act as a potential tumor promoter for different cancer cell types, and support tumor progression and invasiveness through the activation of several intracellular signals. Interaction with the tumor microenvironment and extracellular matrix remodeling, the exosomal release of pro-oncogenic factors as well as the induction of epithelial-mesenchymal transitions are the most investigated mechanisms. Moreover, ASCs have also demonstrated an elective tumor homing capacity and this tumor-targeting capacity makes them a suitable carrier for anti-cancer drug delivery. New genetic and applied nanotechnologies may help to design promising anti-cancer cell-based approaches through the release of loaded intracellular nanoparticles. These new anti-cancer therapies can more effectively target tumor cells, reaching higher local concentrations even in pharmacological sanctuaries, and thus minimizing systemic adverse drug effects. The potential interplay between ASCs and tumors and potential ASCs-based therapeutic approaches are discussed.

## 1. Introduction

In the last decade, the tumor microenvironment has gained more and more relevance in cancer biology and progression [1]. It is characterized by a complex network consisting of different cellular types organized through an intricate signaling system [2]. These signals influence tumor progression and metastatic capacity [3]. Immune cells, adipocytes, myofibroblasts, extracellular matrix, tumor cells, and mesenchymal stem cells (MSCs) can be found in this complex microenvironment [4]. MSCs were first isolated from bone marrow and described by Friedenstein [5] as part of the stroma that supports the maturation of hematopoietic cells. Great efforts have been put into the definition of MSC immunophenotype, and in 2006 the International Society for Cell Therapy (ISCT) proposed a standard panel of surface molecules in order to adequately profile MSCs [6]. According to this consensus, MSCs should be positive for the expression of CD105, CD73 and CD90, and negative for CD45, CD34, CD14 or CD11b, CD79α or CD19 and HLA-DR surface molecules. Adult MSCs were later found in many other tissues [6,7] such as cord blood [8], peripheral blood [9], skeletal muscle [10], dermis [11], synovial membrane [12] and adipose tissue [13]. The presence of cells with stem-like features has also been demonstrated in several cancers, and are identified as a distinct cellular population named cancer stem cells (CSCs) [14]. They present many features in common with mesenchymal cells [15] and authors have speculated about their possible origin from transformed MSCs [16].

Since their discovery in adult human tissues, MSCs have been characterized by several features that are shared by all mesenchymal sub-populations independent of their origin. They include the capacity to adhere to plastic in standard culture conditions, the ability to differentiate into mesenchymal lineages, and the expression of distinct surface markers [5,17]. Even though, MSCs were first isolated from bone marrow, bone marrow-MSCs (BM-MSCs) are a relatively small population. In a bone-marrow aspiration they account for approximately 0.001–0.01% of all the nucleated cells, depending on the technique used [18]. A scarce harvest implies the need for an ex-vivo expansion. This is particularly true for clinical applications that require a large number of cells. For this reason, adipose tissue has been recognized as an optimal source for harvesting MSCs, both for pre-clinical models and for clinical use [19]. Adipose stem cells (ASCs) present numerous advantages compared to BM-MSCs. Their harvesting is less invasive, with a cellular yield more than 1000-fold higher when compared to BM-MSCs and cord-blood MSCs [20,21]. Furthermore, they have a longer life-span, higher proliferative capacity, shorter doubling time and later in vitro senescence compared to BM-MSCs [22]. ASCs were firstly isolated from white adipose tissue and described by Zuk et al. in 2001 [23], then immunophenotypically characterized by Yoshimura et al. in 2006 [24]. ASCs are localized in the perivascular niche and are part of the stromal vascular fraction that is obtained after enzymatic digestion (or mechanical dissociation) and centrifugation of adipose tissue [25]. According to a joint statement of the International Federation for Adipose Therapeutics and Science (IFATS) and of the ISCT, ASCs have been identified phenotypically as positive for CD13, CD29, CD44, CD73, CD90, CD105 while they are negative for CD31 and CD45. Moreover, they are able to proliferate in vitro and to undergo trilinear differentiation toward adipogenic, chondrogenic and osteogenic lineages [26]. ASCs are able to secrete multiple growth factors (GFs) including platelet-derived growth factor (PDGF), vascular endothelial growth factor (VEGF), basic fibroblast growth factor (bFGF), insulin-like growth factor 1, hepatocyte growth factors (HGF), and transforming growth factor (TGF)-β1 [27]. GF secretion is responsible for a pro-angiogenetic and anti-apoptotic effect of ASCs [28]. ASCs are also able to differentiate into endothelial cells that participate in the formation of new vascular structures [29]. Moreover, TGF-β1 secretion promotes an immunomodulatory effect, and increase extra-cellular matrix deposition and collagen organization [30].

All these particular characteristics of ASCs have encouraged their use in many clinical situations, particularly in those tissues where healing is impaired by inadequate blood supply and a chronic inflammatory state, such as in radio-treated tissues [31]. Because many patients treated with ASCs came from oncological settings, the use of ASCs as a possible therapeutic agent has been paralleled by growing concerns about their possible pro-oncogenic risk in residual disease. Concerns have mainly been motivated by their angiogenic, anti-apoptotic and immunomodulatory properties.

The simultaneous presence of noxious and beneficial aspects have earned ASCs their reputation as a “double-edged sword” [32,33]. The relationship between ASCs and cancer has been deeply investigated in preclinical models [34]. Growing evidence has recognized peri-tumor adipose tissue as well as its progenitor cells, including ASCs as a source of pro-tumor factors [35].

ASCs can influence tumor growth, aggressiveness and metastatic sprouting through different pathways. ASCs’ secretion of pro-angiogenetic growth factors and chemokines like PDGF, VEGF c-kit is useful to increase blood supply in poorly vascularized tissues, but at the same time, it could induce the proliferation of endothelial cells and foster the development of a tumor-supporting vascular network, which could lead ultimately to the disease spreading [36,37,38].

ASCs are also able to affect the epithelial to mesenchymal transition (EMT), which is another fundamental step in tumor progression. EMT determines the shift of the tumor toward a more invasive and metastatic phenotype [39]. It has been reported that ASCs can induce EMT in breast cancer cells by acting on multiple pathways, especially through PI3K/AKT signaling and p38 MAP kinase [40,41] or by overexpressing leptin, as shown by ASCs from obese patients [42].

The effects of ASCs on EMT and cellular migration are also mediated by the Wnt pathway [43]. Exosomes secreted by ASCs are able to induce breast cancer cell migration mediated by Wnt-signaling [44]. On the other hand, breast tumor-derived factors are able to promote ASC transformation into tumor-associated fibroblasts, through the inhibition of Wnt signaling [45].

Secretion of TGF-β is also involved in the complex network between stem cells and ASCs. As aforementioned, ASCs release TGF-β, which is responsible for collagen deposition and extra-cellular matrix (ECM) remodeling, a fundamental step in wound healing [30]. However, TGF-β secretion and induction of the TGF-β/SMAD signaling pathway promote EMT in cancer cells [46]. Parallelly, TGF-β signaling is able to induce myofibroblastic differentiation in ASCs exposed to breast cancer exosomes, thus promoting desmoplastic transformation of the tumor microenvironment [47]. TGF- β is also among the main causes of the immunomodulatory effect of ASCs. Immune-mediated response to tumors is impaired by TGF-β1, HGF, IDO, and IFN-γ, which are secreted by ASCs [38,48,49,50,51,52].

ASCs are also able to elicit drug resistance and cell proliferation in the breast cancer cell line MCF-7/ADR (a multidrug-resistant breast cancer cell model) mediated through C-terminal Src kinase (Csk)-binding protein (Cbp) expression [53].

As reported in a mouse model, ASCs can reach the tumor microenvironment even from distant body areas through systemic circulation, thus favoring tumor growth [54].

The tumor itself is able to stimulate ASCs migration and their homing in on the cancer microenvironment. The main mediators of this process are factors like MCP-1 and SDF-1 secreted by cancer cells and inflammatory cells embedded in the tumor stroma [55].

These specific tumor-homing properties of ASCs, even from distant sites, could also be exploited in a therapeutic way. ASCs could be transformed into a “Trojan horse” capable of delivering anti-neoplastic agents directly inside the cancer microenvironment. MSCs have been tested as vectors for several innovative cancer therapies such as drug-loaded nanoparticles, micro-RNAs, viral vectors encoding tumor suppressor genes and many others [56]. However, their easy harvest and higher availability qualifies ASCs as an optimal candidate to be used as a carrier when compared to other MSCs from other sources. Nonetheless, data about the oncological safety of routine use of ASCs in clinical settings are still contradictory. In vivo data does not seem to confirm pre-clinical evidence about a pro-oncogenic role of ASCs. In numerous clinical studies ASC use does not seem to increase the risk of locoregional or distant tumor recurrence. Nevertheless, strong definitive evidence on its oncological safety has not been provided yet. At the moment, this is an important limitation on the possible future use of ASCs as a pharmacological carrier since it could ultimately impact on tumor natural history in a negative way. In this review, we discuss the possible mechanisms underlying ASC–cancer interactions and promoting tumor progression and metastasis, which has been recently reported in experimental models. We also explore new possibilities offered by ASCs as future therapeutic carriers of new anticancer molecules. Finally, we review up-to-date clinical studies using ASCs in oncological patients, in order to evaluate whether ASCs could be considered a safe drug-carrier in oncological settings.

## 2. Interplay between ASCs and Cancer Cells: Mechanisms Underlying Tumor Progression in Experimental Models

### 2.1. The Role of ASCs in the Tumor Microenvironment and Cancer Progression

Different and conflicting data from the literature indicate that ASCs can favor tumor growth and progression. As reported above, among these studies, the hypothesis of a possible interaction of ASCs with tumor microenvironment is the most endorsed. The cross-talk between mesenchymal cells, including ASCs, and cancer cells is not yet fully understood. ASCs are located in perivascular niches contributing to cell turn-over and stem cell homeostasis [57]. Dynamic and reciprocal interactions between epithelial and stromal cells occur during cancer progression by the exchange of cytokines, chemokines and growth factors, which develops a favorable microenvironment for cancer growth [58]. In addition, several secreted factors such as MCP-1 and SDF-1 produced by cancer and inflammatory cells, induce the homing and migration of non-resident ASCs into the tumor microenvironment [55]. Their recruitment into the tumor microenvironment promotes cancer growth, metastasis and stroma formation [59]. Several conflicting pre-clinical results come from studies about the influence of ASCs in cancer progression. Proangiogenic factors and chemokines expressed by ASCs, such as c-Kit, PDGF, VEGF favor endothelial proliferation and neoangiogenesis, thus supporting tumor growth [36,37,38]. As reported, ASCs express the surface marker CD44 that anchors some matrix-metalloproteinases (MMPs). This binding CD44-matrix-MMPs has been demonstrated to influence the ECM reorganization [22]. Moreover, experiments in vivo and in vitro have reported that ASCs favor tumor growth, ECM deposition and neoangiogenesis, and promote the formation of a complex network among fibroblasts together with desmoplastic reactions [60]. The latter, which cause rupture of the basement membrane and an inflammatory remodeling of the ECM, are a stromal response to cancer cell infiltration [61]. This process requires the MMP activity that is increased in vitro by the co-culture of human ASCs and breast cancer cells [62].

It has been reported that ASCs promoted endothelial cell vascular sprouting when embedded within 3D collagen type I hydrogel. These data indicate the angiogenic capability of ASCs [63]. This phenomenon has also been reported in epithelial ovarian cancer (EOC) cells, whose proliferation and invasion were promoted by the ASC co-culture, which induced the secretion of high MMP levels [64]. Enhanced EOC growth and metastatic potential were also found in mouse xenografts, mediated by an increase in MMP2 and MMP9 expression [64]. The same increase in MMP activity, tumor growth and invasion was obtained through a ASC co-culture with osteosarcoma (OS) tumor cells via STAT3 activation [65]. These results were also confirmed in a mouse model of osteosarcoma, in which human ASCs promoted STAT3 activity, eliciting tumor proliferation, invasion and metastasis [66]. The effect of ASCs on ECM remodeling has been reported to be dependent on the donor’s obesity status and on the cell harvesting site [67]. In fact, ASCs isolated from the subcutaneous abdominal adipose tissue of obese patients demonstrated an increased invasion through Matrigel as well as through a chick chorioallantoic membrane. This effect depended on calpain-4, calpastatin, and MMP15 activity [68]. The co-culture of ASCs with melanoma cell lines significantly increased tumor migration and invasion capacity [38]. The analysis of gene expression in co-cultures has highlighted an increase in the expression of different tumor-promoting genes such as CXCL12, PTGS2, IL-6, and HGF, as well as the upregulation of numerous tumor-associated proteins, e.g., several pro-angiogenic factors such as VEGF, IL-8, CCL2, and of different MMPs, especially MMP2 [38].

### 2.2. Exosome Releasing

Recently, particular attention has been paid to exosomes, since it is known that they contribute to the paracrine effects of MSCs [69]. Exosomes are small, intraluminal vesicles (<100 nm) released by their fusion with the plasma membrane [70]. They are secreted by cells in order to exert regulatory functions and to release bioactive molecules such as RNA, DNA and enzymes [71]. The importance of ASC-secreted exosomes in cancer biology has been recently reported. Seo et al. described the inhibitory effect of exosomal miR-503-3p from ASC-conditioned medium on breast cancer cell proliferation, and the self-renewal of cancer stem cells (CSC). These miRNAs downregulate the expression of cancer stemness markers. In breast cancer xenografts, tumor growth was counteracted by the presence of miR-503-3p, supporting the role of this miRNA as a specific CSC inhibitor [72]. ASC exosomes affected tumor grading and growth in rats with N1S1-induced hepatocellular carcinoma (HCC) and increased the number of circulating and intra-tumoral natural killer T (NKT) cells, thus demonstrating the anti-cancer immunomodulatory function of ASC exosomes [73]. Exosomes from ASC conditioned medium showed an inhibitory effect on ovarian tumor cells A2780 and SKOV-3, decreasing cancer growth, migration and colony formation [74]. In addition, exosomes from ASC-conditioned medium induced cancer cell apoptosis by the upregulation of pro-apoptotic genes and downregulation of the anti-apoptotic BCL2. In fact, by sequencing exosomal RNAs, a rich population of miRNAs with anti-cancer activities has been identified [74]. ASC-conditioned medium inhibited the proliferation of metastatic prostate cancer (PCa) by induction of cell apoptosis through exosomal miR-145, whose knockdown reverted the anti-tumor effect of ASC-conditioned medium [75]. However, some authors reported that ASC exosomes favored breast cancer cells invasion through Wnt signaling [44].

Data from the literature has reported conflicting results about the role of ASCs in glioma and glioblastoma behavior [76,77,78,79]. In particular, it has been demonstrated that ASC-conditioned medium promoted the epithelial-to-mesenchymal-like transition in glioma cells in vitro [76] as well as the migration of glioblastoma cells [77], likely due to tumor release of migration-promoting chemokines [78]. A study by Yang et al. reported the pro-apoptotic activity of ASC-conditioned medium on U251 glioma cell culture, instead [79]. However, a study on U87MG glioblastoma cells showed that ASC exosomes, when up-taken by tumor cells were not effective in tumor growth inhibition [80].

Several conditions could influence the interaction between ASCs and cancer cells; in particular, cancer origin and histotype, as well as different treatment protocols (e.g., ASCs/cancer cells ratio, injection modality, kinetics of carcinogenesis) can potentially affect standardization [50]. However, it is commonly agreed that cancer and inflammatory cells release molecules that induce ASC homing and migration into tumor microenvironment [55].

### 2.3. Functional Changes in ASCs Induced by Cancer Cells

Even though ASCs can influence tumor tropism, some researchers have postulated the existence of a bidirectional effect. According to this hypothesis, the tumor exerts a paracrine effect on ASCs, thus determining phenotypic and functional changes in these cells. As previously reported, ASCs in co-culture with H358 lung cancer cells differentiate into myofibroblasts [81]. The same myofibroblastic differentiation is demonstrated in ASCs exposed to breast cancer exosomes [47] and to breast tumor-derived factors [45]. In addition, breast cancer-derived exosomes seem to induce the myofibroblastic phenotype of ASCs via the SMAD-mediated signaling pathway [82]. Furthermore, exosomes from ovarian cancer cells stimulate ASC transformation into tumor-supporting myofibroblasts [83].

It has been reported that ASCs isolated from sub-abdominal adipose tissue of patients with urological neoplasms show similar growth kinetics, equivalent mesenchymal surface markers and differentiation potential similar to ASCs from adipose tissue of age-matched non-oncogenic participants [84]. Molecular karyotyping of expanded patient ASCs did not show alterations related to the oncological disease. In addition, exosomes show equivalent miRNA content from both cancer patients and from non-oncogenic participants, thus indicating a possible use for autologous stem cell transplantation in clinical settings [84]. However, in a study conducted on prostate cancer patients, ASCs primed with prostate cancer cell-conditioned medium formed prostate-like neoplastic lesions in vivo and reproduced aggressive tumors in secondary recipients [85]. Moreover, primed ASCs acquire cytogenetic aberrations and EMT, expressing epithelial, neoplastic, and vasculogenic markers similar to prostate tumor xenografts. The authors postulate that the tumorigenic reprogramming of ASCs is due to the oncogenic factors contained in prostate cancer cell-derived exosomes, including H-ras and K-ras transcripts, oncomiRNAs miR-125b, miR-130b, and miR-155 as well as the Ras superfamily of GTPases Rab1a, Rab1b, and Rab11a [85]. Wang et al. report that lung cancer exosomes could be internalized by ASCs, significantly inhibiting their adipogenesis and adipogenic-specific genes through a TGFβ-mediated signaling pathway [86].

## 3. ASCs as Anti-Tumor Agent Carriers: A “Trojan Horse” Against Cancer Progression

**Figure 1:**
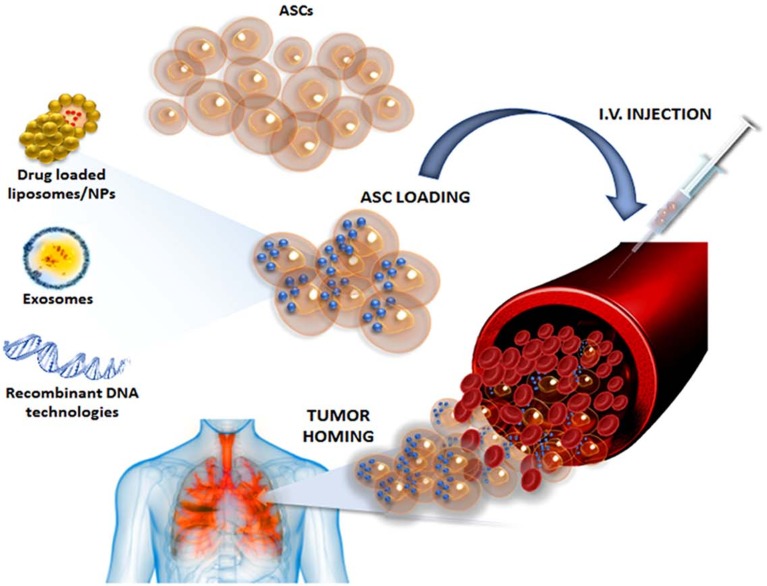
A schematic representation of new anti-cancer strategies based on adipose-derived stem cell delivery systems.

Recent preclinical studies based on the ability of MSC to home to the tumor microenvironment suggested their use as anti-cancer drug delivery carriers [87,88,89] (Figure 1). In particular, it has been reported that MSCs can uptake and subsequently slowly release Paclitaxel (PTX) through exosomes, inhibiting the proliferation of leukemia, multiple myeloma, mesothelioma, osteosarcoma, prostatic carcinoma and neuroblastoma [87,88,89,90,91,92]. PTX is a widely used chemotherapeutic drug that acts as a microtubule-stabilizing agent, blocking cancer cell mitosis [93]. As reported by Scioli et al., ASCs can uptake and release PTX with no significant effects on their viability and cell cycle. PTX-loaded ASCs as well as their conditioned medium strongly inhibit CG5 breast cancer survival and proliferation both in vitro and in vivo [94]. Based on this MSC ability, Wu Jet al. demonstrated that gold nanorod embedded hollow periodic mesoporous organosilica nanospheres (GNR@HPMOs) show high PTX loading capability, excellent photothermal transfer ability upon near-infrared (NIR) light irradiation, and are well-retained by MSCs after internalization without affecting their viability and tumor-homing capability [95]. In vitro experiments have revealed that GNR@HPMOs-PTX-loaded MSCs have synergistic chemo-photothermal killing effects on breast cancer cells and significantly inhibit tumor growth in vivo [95]. These data offer new perspectives concerning the use of ASCs during breast reconstruction as an additional and synergistic local therapy against tumor relapse.

The therapeutic use of ASCs in brain tumors is currently under thorough investigation. Unfortunately, these tumors are often found in surgically inaccessible areas and anti-cancer drugs have to cross the blood-brain barrier to reach them. To this end, some researchers are studying the efficacy of ASCs primed with nanotherapeutic payloads for thermo/chemotherapy on brain tumors [96]. The nanoparticle (NP) payload is obtained from the co-assembly of poly (γ-glutamic acid-co-distearyl γ-glutamate) with poly (lactic-co-glycolic acid), PTX, and oleic acid-coated superparamagnetic iron oxide NPs. ASCs loaded with NPs show good viability, even at high PTX concentration (30 μM), and good dispersion and distribution in tumor tissue. This ASC-based approach combined with high frequency magnetic field has proven to be significantly effective, with a 4-fold increase in therapeutic index on brain astrocytoma (ALTS1C1)-bearing mice compared to typical chemotherapy using temozolomide [96].

A promising strategy against brain tumors is also represented by ASC-based therapeutic gene delivery. Exploiting the intrinsically higher TGF-β expression in glioblastoma than in normal brain tissue, some researchers have engineered human ASCs in order to increase the expression of TRAIL under the trigger of TGF-β signaling via a SMAD4-controlled minimal promoter [97]. The therapeutic efficacy was proven by in vitro and in vivo assays using primary patient-derived glioblastoma models, which decreased tumor volume and prolonged survival time, as well as limiting off-target cytotoxicity with the controlled expression of the suicide inductor TRAIL [97]. The same strategy has proven effective in a mouse model of brainstem glioma [98]. In a study by Li et al., ASCs were pre-exposed to TGF-β before cell transfection with lentiviral vector containing TRAIL, in order to enhance their homing to glioblastoma by increasing the expression of CXC chemokine receptor 4 (CXCR4) [99]. Genetically engineered ASCs overexpressing TNFα are able to induce apoptosis via caspase 3/7 activation in human breast cancer cells and melanoma xenograft, ovarian cancer cells, glioblastoma, and to a lesser extent, on the colon [100]. Moreover, human ASCs genetically modified to express interferon γ-induced protein 10 (IP-10), a potent chemoattractant with antitumor activity, have proven effective in the treatment of lung metastasis in an immunocompetent mouse model of metastatic melanoma [101].

Another possible strategy, based on gene-directed enzyme prodrug therapy, consists of the transfection of bacterial and/or yeast cytosine deaminase (CD) enzyme, which converts the far less toxic substrate 5-fluorocytosine (5-FC) to highly toxic 5-fluorouracil (5-FU) [102]. CD expression sensitizes transfected ASCs to the 5-FC at its highest concentration only, unlike cancer cells that are much more sensitive, suggesting the existence of an endogenous mechanism for ASCs which might be able to eliminate the suicide effect of transgene expression (exosome release?) [102]. Therefore, the co-administration of 5-FC with transfected ASCs has been proven to be an effective therapy for colon cancer micro-metastasis as well as glioblastoma and melanoma [102,103,104]. Based on the same principle, herpes simplex virus-thymidine kinase (HSV-tk) expressing ASCs (TK-ASC) exert cytotoxic effects on glioblastoma cells upon treatment with prodrug ganciclovir [105]. Lu et al. engineered ASCs with a modified E6/E7 antigen (non-oncogenic protein), derived from human papillomavirus type 16 in order to deliver this antigen and elicit an anti-tumor immune reaction, as demonstrated through the response of CD4^+^ T, CD8^+^ T and NK cells in mice bearing colon and lung cancer [106]. However, there is a need to enable non-viral nanobiotechnology in order to allow safe and effective gene therapy. In a study conducted by Mangraviti et al., the authors demonstrated the possibility of using non-viral, biodegradable polymeric nanoparticles (NPs) containing a BMP4-expressing DNA plasmid, in order to engineer human ASCs both with a higher efficacy (75% of cells) compared to leading commercially available reagents and with a high cell viability to preserve ASC migration and invasion capacities. NP-engineered ASCs are able to deliver BMP4, which targets human brain tumor initiating cells (BTIC), a source of cancer recurrence, in a human primary malignant glioma model [107]. In a study conducted by Jiang et al., human ASCs show a more robust TRAIL expression through a polymeric nanoparticle-mediated non-viral transfection. Engineered ASCs effectively induce tumor-specific apoptosis and exhibit long-range directional migration and infiltration toward a xenograft from patient-derived glioblastoma cells in a mouse model, extending survival and reducing the occurrence of microsatellites [108].

ASCs represent the best candidates for exosome-wrapped miRNA strategy because they can release large amounts of exosomes [109]. Since glioma cells and glioma stem cells (GSCs), a small subpopulation of cancer stem cells implicated in therapeutic resistance and tumor recurrence, express very low levels of miR-124 and miR-145, Lee et al. successfully tried to deliver these miRNA mimics in glioma cells and GSC co-cultures through ASC exosomes. Their internalization via gap junction-dependent and independent processes determines a decrease in their respective target genes, SCP-1 and Sox2, reducing glioma cell migration and GSC self-renewal. Moreover, when administered intracranially, ASCs are able to deliver miR-124 mimic to glioma xenografts [110].

As reported by Lou et al., ASCs transfected with a miR-122 expression plasmid are able to deliver miR-122 through their exosomes, affecting cell viability, apoptosis, and the cell cycle of hepatocellular carcinoma (HCC) cells. In addition, ASCs transfected with miR-122 also sensitize HCC xenograft to sorafenib in vivo. It is known that HCC displays a high resistance to conventional chemotherapy and miR-122 has proven essential to promote chemosensitivity, therefore it represents a valid tool for a targeted strategy [111].

The pro-apoptotic activity of NK cell-differentiated ASCs transfected with miR-150 on pancreatic cancer cells PANC1 has also been reported [112]. MiR-150 is responsible for the development and activation of NK cells as well as their production of IFNγ, and this strategy produces effective immunomodulatory activity [112]. CSCs are a small cellular population found in tumors that show stem cell-like properties and influence tumor progression, metastasis, and drug resistance. In a study by Lee et al., the authors speculate on a possible anti-cancer therapy based on reprogramming CSC into non-tumorigenic cells using ASC exosomes. According to this study, exosomes from osteogenic differentiated human ASCs, containing specific cargos with osteoinductive properties, successfully induced CSCs to express osteogenic-related genes such as alkaline phosphatase, osteocalcin, and runt-related transcription factor 2. In addition, the differentiation decreases some drug-resistance genes such as ATP binding cassette transporter, the breast cancer gene family (BCRA1 and BCRA2), and the ErbB gene family [113]. A schematic summary of the aforementioned new anti-cancer strategies based on ASC delivery systems is reported in Table 1.

Although ASCs have been proven to be good carriers for anti-cancer drug delivery, several issues remain to be clarified. ASCs’ ability to home to and interact with the tumor microenvironment represents a double-edged sword. The clinical safety of ASC-based therapies represents an important question that remains open. The fate of these cells once they have reached the tumor site and released anti-cancer drugs is unknown. Studies in the literature mainly draw their evidence from pre-clinical models and the few clinical studies that have been undertaken (particularly in the field of regenerative medicine) have used native ASCs with no manipulation.

## 4. Oncological Safety of ASC Clinical Applications

As highlighted above, in vitro and in vivo evidence associates ASC activity with enhanced tumor cell proliferation rate and the cancer shifting to a more invasive and aggressive phenotype [34]. Although these data seem to outline a general trend towards the pro-carcinogenic role of ASCs, clinical reports do not appear to confirm laboratory studies [2]. Wide availability, easy harvesting and minor morbidity on donor sites are the major strengths in favor of ASC use over other types of MSCs in regenerative medicine. They are a useful tool in many clinical settings, both oncological and non-oncological [114]. Their pro-angiogenic capacity and their ability to differentiate into multiple cell types make them suitable to cure radiotherapy-induced tissue damage and to treat soft tissue defects in difficult areas like those that have undergone oncological treatments [115].

The use of ASC-based treatments has been tested in several oncologic settings ranging from radiotherapy-induced xerostomia [116] to osteosarcoma defects [117,118], from soft tissue reconstruction after sarcoma resection [119] to laser assisted pulmonary metastasectomy [120] with promising results. Nonetheless, many of these are initial, single center experiences with retrospective data only, small numbers, short follow up times and low quality evidence. However, in post-oncologic breast reconstruction, autologous fat grafts has been among the most commonly performed surgical techniques during the last decade and have been useful across a wide clinical spectrum from post quadrantectomy defects to total breast reconstructions [89].

Most of the data about the oncological safety of autologous fat grafts and ASC use come mainly from a subgroup of patients represented by female patients affected by breast cancer. Fat grafting was described by Coleman in 1997 [121] and since then it has been widely used for aesthetic and reconstructive purposes including oncological breast surgery. The American Society of Plastic Surgeons (ASPS) formed a task force in 2007, which concluded in 2009 that fat grafting was safe with no malignancy risk [122]. Nonetheless, in 2011 a possible malignancy recurrence risk underlying autologous lipofilling and ASCs was hypothesized again in a joint statement by the American Society for Aesthetic Plastic Surgery and ASPS [123]. In 2015, the ASPS released a grade B recommendation stating that fat grafting does not increase the risk for local recurrence even though strict adherence to radiological follow-up protocols and an adequate disease-free interval are mandatory (https://www.plasticsurgery.org/Documents/Health-Policy/Principles/principle-2015-post-mastectomy-fat-grafting.pdf). Hence, several clinical studies have investigated the impact of fat grafting on the local and distant recurrence of cancer.

An overview of data from the literature concerning fat grafting in oncological patients is shown in Table 2. Initially, the safety evaluation of fat grafting was conducted retrospectively on populations including both oncological and non-oncological patients [124,125].

In 2010, Rigotti et al. published a retrospective study on 137 mastectomy patients from a population of 911 patients that included 104 breast conservative surgeries (BCS) and 807 mastectomy cases (MST) with a median follow-up of 7.6 years after transplantation. There was no control group and the same patients served as controls where the follow up was split into pre- and post-fat grafting groups [126]. The local recurrence (LR) rate was similar in both groups, suggesting that there was no any additional risk related to fat grafting. The study design was controversial and no data about distant metastases (DM) and tumor phenotype were reported [126].

In 2012 a French/Italian multicentric retrospective study [127] enrolled 321 patients (196 MST and 125 BCS) with both invasive and in-situ cancer. They matched a control group (not grafted) with the same demographic and oncological features with a ratio case/control of 1:2. The median follow up was 26 months after fat grafting (cases) or demolitive surgery (control group). Local and distant recurrence rates were similar in both groups (2.5% LR for the cases vs. 3% for the control group; 4.6% DM for the cases vs. 4.7% for the control group). Nonetheless, in the subgroup analysis, the group of fat grafting patients with in-situ cancer had a significantly increased risk for LR. The same investigators tried to evaluate fat grafting in this potentially at-risk population with another retrospective matched-cohort study focusing only on patients with intra-epithelial neoplasia [129]. A total of 59 patients and 118 controls were considered for a median follow up time of 38 and 42 months from fat grafting procedures, respectively. The LR rate was significantly higher in the case group (18% for cases vs. 3% for control). Women < 50 years old with high-grade neoplasia, high levels of Ki-67 and who had undergone quadrantectomy had the higher risk. Even though this study had several limitations, including its retrospective design, it was the first to demonstrate a possible oncological risk behind fat grafting in specific patient subsets.

The RESTORE2 trial was one of the few multicentric prospective trials to evaluate the safety of fat grafting enriched with ASCs [128]. Sixty-seven women with a history of BCS were enrolled and treated with fat grafting enriched with ASCs. The follow up was 12 months. Even though no cases LR were found, the follow up time was too short to determine the oncological risk of the procedure.

In 2015, Kronowitz et al. published a large retrospective matched controlled study which enrolled 719 patients (79 BCS and 639 MST) treated with fat grafting and a control group of 670 non-grafted patients (73 BCS and 591 MST) with a mean follow up of 60 months and 44 months, respectively [131]. LR and DM rate were similar between groups (1.3% LR for cases vs. 2.4% for controls; 2.4% DM for cases vs. 3.6% for controls), although fat grafting seemed to significantly increase the risk for LR in a hormonal therapy subgroup (1.4% in fat grafting vs. 0.5 % in controls). Although not statistically significant, a trend toward increased LR in patients who had undergone fat grafting with high-risk features, i.e., advanced tumor stage or neoadjuvant therapy, was noticeable [135]. Several clinical and pathological tumor features were not matched between cases and controls, which constitutes an important limitation of this study, and an increased risk of bias. Gale et al. in 2015, retrospectively evaluated 211 cases with 2:1 matched controls (*n* = 422). The mean follow up was 32 months. No significant differences in terms of local, loco-regional or distant recurrences were described between groups (0.95% LR in cases vs. 1.90% in controls; 3.32% DM in cases vs. 2.61% in controls). Therefore, there was no evidence of increased oncological risk derived from fat grafting [130]. A single center study with a matched retrospective case-control design analyzed the oncological impact of fat grafting in 100 breast cancer patients reconstructed with a deep inferior epigastric artery perforator (DIEP) microsurgical flap [134]. Controls were matched 1:1 and the median follow up time was 31 months. Patients who underwent fat grafting had a disease-free survival similar to controls. The overall recurrence rate was 12% for fat grafting and 13% for controls. Interestingly, a significantly increased risk of recurrence occurred in fat grafting subgroups: in women with positive nodal status and a high-grade neoplasia. According to the authors, the increased risk could be explained by growth factors released from ASCs that reactivated dormant cancer cells in nodal occult micro-metastasis.

In 2107, Cohen et al. published a case-control study comparing two groups of patients who underwent MST with or without fat grafting (fat grafting, *n* = 248; not grafted control, *n* = 581). The incidence of LR (2.5% fat grafting vs. 1.9% control) and of DM (1.9% fat grafting vs. 3.1% control) were not significantly different between groups. The fat grafting group included patients with lower-grade neoplasia and lower pathologic cancer staging with a possible selection bias [133]. Moreover, in 2017, a multicentric case-cohort study was reported by Myckatyn et al. [132]. The study population included patients retrospectively selected with invasive ductal carcinoma from stage I to III and a history of MST and immediate breast reconstruction. Among 3271 eligible patients from four institutions, 1197 patients were enrolled. A cohort of 225 recurrences (55% distant, 11% regional, and 34% local) was compared to a randomly selected control group consisting of 972 patients (30% of the entire study population). In a bivariate analysis, fat grafting status (fat grafting yes vs. no) was not associated with a reduced time to recurrence. Even on a multivariate analysis adjusting the model for age, stage, HER2/neu-positive, estrogen receptor-positive, and body mass index, fat grafting patients did not report an increased risk of recurrence. The authors concluded that fat grafting does not increase the risk of cancer recurrence for patients who underwent MST for an invasive ductal carcinoma from stage I to III.

A phase-3 randomized controlled clinical trial concerning fat grafting safety was registered by a French group. Enrollment started in 2010 but proceeded slowly with fewer numbers than expected (196 recruited patients vs. 440 expected cases). The study was completed in February 2017 but the results have not been published yet (clinicaltrials.gov NCT01035268).

A meta-analysis in 2015 examined 2428 oncological patients from 35 studies with a median follow up of 22.6 months [136]. The recurrence rate was 5.7% for the fat grafting cases and 4.7% for the control group, showing no significant results. However, the heterogeneity of these studies has raised some critical issues.

Another meta-analysis study conducted by Wazir et al. pooled 2382 MST and BCS patients with a mean follow up of 36.2 months for the MST and 30.2 for BCS. A non-significant difference in mean locoregional recurrence rate between groups was found: 3.2% for lipofilling compared to 4% for patients without lipofilling [137]. According to this meta-analysis, lipofilling should be considered a safe procedure when used in oncological breast reconstruction.

In 2016, a meta-analysis by Groen et al. evaluated fat grafting safety in oncoplastic breast reconstruction in 1371 patients from 14 studies with a follow up time from 12 to 36 months. A local recurrence rate of 2.5% and a distant recurrence rate of 2% was found in fat grafting patients with no difference between MST and BCS [138]. These rates, according to the authors, are similar to those reported for patients who never underwent fat grafting. However, the authors reported high heterogeneity in the studies without providing solid evidence.

Several systematic reviews on fat grafting safety have been carried out [2,139,140]. They all conclude that fat grafting can be a safe option for oncological patients but further studies and more robust evidence is needed.

Fat grafting appears to be safe for patients with a history of MST, while in BCS patients, especially in high-risk categories, more data should be provided. DMs are not reported in many studies, with the loco-regional recurrence being the focus. Nonetheless, preclinical studies support the role of ASCs as promoters of EMT and invasiveness, increasing the risk of tumor spreading [39,40,41,42,47,81].

Further studies are needed to evaluate distant recurrences more carefully before being able to declare the use of ASCs to be completely safe. More prospective, multicentric, randomized clinical studies with longer follow ups are required for more solid results and for detecting the potential risks underlying ASC clinical use and the specific at-risk categories of patients. These issues concerning the oncological safety of ASCs should definitely be clarified, especially because ASCs are used more and more often in many oncological settings and are potential carriers for new anticancer drug-delivery systems in the relatively-near future.

## 5. Conclusions and Future Perspectives

Different and conflicting data from the literature indicate that ASCs can favor tumor growth and progression. A possible explanation for these conflicting results can be found in the multiplicity of factors that influence the interaction between ASCs and cancer cells, such as their origin and pre-treatments, cancer type and different experimental conditions, which may affect standardization. However, the interaction of ASCs with the tumor microenvironment has been clearly demonstrated. The precise mechanisms related to cancer invasion and metastasis are far from being completely defined. This is mainly due to the complexity of the molecular pathways involved and to the limitations of preclinical investigation on tumor evolution. Appropriate models considering not only tumor cells, but also the surrounding microenvironment should be developed for this purpose.

On the other hand, the tumor-homing properties of ASCs could also be exploited in a therapeutic way. ASCs could be the “Trojan horse” that delivers anti-neoplastic agents directly into the cancer microenvironment, especially in the case of tumors located in surgically inaccessible areas and pharmacological sanctuaries. In this sense, ASC-based therapies represent a promising strategy against brain tumors. ASCs have been tested in preclinical models as vectors for several innovative cancer therapies such as drug-loaded exosomes and nanoparticles, micro-RNAs, viral vectors encoding tumor suppressor genes and many others.

Nevertheless, up-to-date clinical data does not allow strong statements about the safety of ASC use in cancer patients. Further studies with more robust evidence are needed to clarify the oncological safety of ASC-based therapies in order to fully exploit their encouraging potential in cancer treatment and to translate these results in clinical settings.

## Figures and Tables

**Table 1:** New anti-cancer therapeutic strategies based on ASC delivery systems.

Category	Type	Mechanism of Action	Target	References
Drug loading	PTX	anti-mitotic activity	human leukaemia MOLT-4 cells human osteosarcoma SK-ES-I cells human prostatic carcinoma DU145 cells human neuroblastoma GI-LI-N and SH-SY5Y human breast cancer CG5 cells	[89] [89] [89] [89] [94]
Drug-loaded NPs	PTX	anti-mitotic activity + high frequency magnetic field	murine brain astrocytoma ALTS1C1	[96]
Gene delivery by viral vectors	TRAIL overexpression	suicide inductor	primary patient-derived glioblastoma human glioma U-87MG primary patient-derived glioblastoma	[97] [98] [99]
	TNF-α overexpression	apoptotic activity	human breast cancer SKBR3 cells human melanoma A375 cells	[110]
	IP-10 overexpression	antitumor activity	murine metastatic melanoma	[101]
	cytosine deaminase	cytotoxicity to 5-fluorouracil	human colon cancer HT-29 cells rattus brain glioma C6 cells human melanoma A375 cells	[102] [103] [104]
	thymidine kinase	cytotoxicity to ganciclovir	human glioblastoma cells 8-MG-BA, 42-MG-BA and U-118 MG	[105]
	modified E6/E7 antigen	immunomodulatory activity	murine lung carcinoma LLC1 cells murine colon carcinoma CT26 cells	[106]
Gene delivery by non-viral vectors	BMP4 plasmid-loaded NPs	antitumor activity	primary patient-derived glioma	[107]
	TRAIL plasmid-loaded NPs	suicide inductor	primary patient-derived glioblastoma	[108]
Micro-RNA transfection	miR-124 e miR-145	reducing cell migration and self-renewal	primary patient-derived glioma and glioma stem cells	[110]
	miR-122	apoptotic activity	human hepatocellular carcinoma HepG2 cells	[111]
	miR-150	immunomodulatory activity	human pancreatic cancer cells PANC1	[112]

Abbreviations: LR, local recurrence; DM, distant metastasis; HT, hormonal therapy.

**Table 2:** Clinical studies on oncological safety using fat grafting.

Clinical study	Year	Design of the Study	Control	N° Patients	Median Follow-Up (Months)	Results with fat Grafting	Reference
Osteosarcoma and soft tissue sarcomas	2015	Retrospective case series	no	17	32.5	LR and DM not reported	[118]
	2018	Retrospective case series	no	60	28.6	not increased recurrence risk (LR and DM)	[119]
Radiotherapy induced xerosthomia	2018	Randomized placebo controlled phase I/II	yes	30	4	LR and DM not reported	[116]
Pulmonary metastasis	2017	Prospective cohort study	yes	40	61	LR and DM not reported	[120]
Breast cancer	2010	Retrospective cohort	no	137	91	not increased LR, DM not reported	[126]
	2012	Retrospective matched cohort	yes	321	26	not increased recurrence risk, higher risk of LR for in-situ cancer subgroup	[127]
	2012	Prospective single-arm trial	no	67	12	no LR, DM not reported	[128]
	2013	Retrospective matched cohort	yes	59	42	increased LR for in-situ cancer patients, DM not reported	[129]
	2015	Retrospective matched cohort	yes	211	32	not increased recurrence risk (LR and DM)	[130]
	2016	Retrospective matched cohort	yes	719	60	not increased recurrence risk, higher risk of LR for HT subgroup	[131]
	2017	Retrospective case-control	yes	225	/	cases are cancer recurrence (2006-2011), not increased recurrence risk	[132]
	2017	Retrospective cohort	yes	248	45.6	not increased recurrence risk (LR and DM)	[133]
	2017	Retrospective matched cohort	yes	100	31	increased LR for positive nodal status and high-grade neoplasia, DM not reported, similar disease-free survival and overall recurrence	[134]

Abbreviations: NPs, nanoparticles.
